# ECONOMICS OF LONG-TERM SERVICES AND SUPPORTS: PRODUCTION, DEMAND, AND COSTS

**DOI:** 10.1093/geroni/igad104.0119

**Published:** 2023-12-21

**Authors:** Christine Bishop

## Abstract

Economic analysis of the production, demand, and utilization of long-term services and supports (LTSS) can generate insights for public policy. This symposium presents a range of economics approaches to LTSS, with policy implications. The first paper considers in-home care as a production process, estimating the impact of various mixes of informal care and paid formal care on health outcomes. The two types of care, and their combination, contribute to different aspects of self-reported health. Paper Two uses the disruption of the pandemic to examine the productivity of an understudied resource in nursing home care provision: informal care by visiting family and friends. The finding that continuing resident interaction with informal caregivers reduced mortality should inspire future research on this valuable and understudied input into nursing home care. A third paper tracks rising wages for nursing staff wages and increasing use of contract nursing, fueled by workforce shortfalls. These trends have consequences for quality, worker attachment, and costs. An exogenous policy change raising the out-of-pocket price to some consumers of nursing home services in Germany enables the fourth paper to estimate elasticity of demand. Individuals with lower levels of need were more sensitive to rising prices than those with greater needs and fewer alternatives. The fifth paper examines the relationship of an Alzheimer’s diagnosis to individual health expenditures in Mexico and for persons of Mexican origin in the US. Cross-national comparisons and urban-rural differences suggest insights into how LTSS for cognitive disabilities may combine with health services in the two countries.

